# Genetic diversity and structure of *Elymus tangutorum* accessions from western China as unraveled by AFLP markers

**DOI:** 10.1186/s41065-019-0082-z

**Published:** 2019-01-29

**Authors:** Wen-Dan Wu, Wen-Hui Liu, Ming Sun, Ji-Qiong Zhou, Wei Liu, Cheng-Lin Zhang, Xing-Quan Zhang, Yan Peng, Lin-Kai Huang, Xiao Ma

**Affiliations:** 1 Department of Grassland Science, Animal Science and Technology College, Sichuan Agricultural University, Chengdu, 611130 Sichuan China; 2 Key Laboratory of Superior Forage Germplasm in the Qinghai-Tibetan Plateau, Qinghai Academy of Animal Science and Veterinary Medicine, Xining, 81108 China

**Keywords:** *Elymus tangutorum*, AFLPs, Genetic diversity, Geographic groups, Eco-environmental factors

## Abstract

**Background:**

Understanding genetic diversity of wild plant germplasm and the relationships between ecogeographic and genetic characteristics may provide insights for better utilizing and conserving genetic resources. *Elymus tangutorum* (Nevski) Hand.-Mazz, a cool-season hexaploid perennial, is an important pasture bunchgrass species used for forages and grassland restoration in Qinghai-Tibet Plateau and northwest China. In this study, 27 *E. tangutorum* accessions from diverse origins of western China were evaluated using AFLP markers in an effort to delve into the genetic relationships among them. The effects of eco-environmental factors and geographical isolation on the genetic diversity and population structure were also elucidated.

**Results:**

On account of 554 polymorphic fragments amplified with 14 primer combinations, the mean values of some marker parameters including polymorphic information content, resolving power and marker index were 0.2504, 14.10 and 23.07, respectively, validating the high efficiency and reliability of the markers selected. Genetic dissimilarity index values among accessions ranged from 0.1024 to 0.7137 with a mean of 0.2773. STRUCTURE, UPGMA clustering and PCoA analyses showed that all accessions could be divided into the three main clusters; however, this results do not exactly coincide with geographic groups. We found medium differentiation (*F*_*ST*_ = 0.162) between Qinghai-Tibet Plateau (QTP) and Xinjiang (XJC), and high differentiation (*F*_*ST*_ = 0.188) among three Bayesian subgroups. A significant correlation (*r* = 0.312) between genetic and geographical distance was observed by Mantel test in the species level, while the weak correlation was detected between genetic and environmental distance for all accessions and most of geographical groups. In addition, a significant ecological influence of average annual precipitation on genetic distance was revealed in XJC group and the Bayesian subgroup A.

**Conclusion:**

This study indicates that AFLP technique are a useful tool to measure genetic diversity in *E. tangutorum*, showing that geographical and environmental factors (especially precipitation) together, play a crucial role in genetic differentiation patterns. These findings underline the importance of local adaptation in shaping patterns of genetic variability and population structure in *E. tangutorum* germplasm collected in Western China.

**Electronic supplementary material:**

The online version of this article (10.1186/s41065-019-0082-z) contains supplementary material, which is available to authorized users.

## Background

*Elymus* Linn. is the largest and most widely distributed genus in the tribe Triticeae, with 150 species grown in temperate regions of the world [1]. This genus has a closer phylogenetic relationship with some of the important cereal crops, such as wheat, barley, rye and triticale [1]. Therefore, it may serve as a valuable natural gene pool of desirable traits for the improvement of these crops [2, 3]. Besides, many *Elymus* species are also used as fodder grasses and ecological protection [1]. *Elymus tangutorum* (Nevski) Hand.-Mazz, a perennial hexaploid species with the StYH genome (2n = 6x = 42), along with *E. duhuricus* Turcz. ex Griseb., *E. excelsus* Turcz. ex Griseb. and *E. ivoroschilowii* Probat. constitute the *E. dahuricus* complex [4, 5]. *E. tangutorum* differs morphologically from *E. dahuricus* Turcz. in the light of the short upward awns and its distribution in the Qinghai-Tibet Plateau (QTP), Xinjiang province of China and the Alpine regions of Central Asia. In QTP, *E. tangutorum* is widely used for the degraded grassland restoration, due to high productivity, drought resistance, cold tolerance, and adaptability [6]. As one of the more important forage grasses in QTP, *E. tangutorum* has been widely studied. Most of studies mainly focused on the domestication, cultivation, phylogenesis and genome constitution [4, 6].

Understanding genetic diversity of wild plant germplasm and the relationships between ecogeographic and genetic characteristics may provide insights for better utilizing and conserving genetic resources [7]. Investigation of germplasm diversity can be implemented via morphological and molecular means. Previous analyses based on agro-morphological characters and geographical origin indicated that a wide range of phenotypic divergence occurred among *Elymus* accessions of ecotypes, and/or cultivars [8, 9]. DNA-based markers have been regarded as practical tools with high efficiency and wide genome coverage in illuminating the pattern of genetic diversity and phylogenetic relationships in plant germplasm resources, with various advantages over phenotypic traits such as being not subject to environmental influences, potential of unlimited numbers, and assay from any development stage. In *Elymus* species, diverse PCR-based markers, employed in diversity and phylogeny studies, such as RAPD [10], ISSR [11], SRAP [12], ScoT [9, 13], and AFLP [14–16] have become prevalent as any prior sequence information is not required. Among different marker systems available at present, AFLP method could explore variation throughout the entire genome, including both coding and non-coding DNA regions and may therefore genome-wide variation was allowed. Moreover, AFLP offers high reproducibility and multilocus polymorphisms simultaneously identified using a single assay. These characteristics make the AFLPs extremely appropriate for molecular characterization of germplasm collection. Although previous work has provided preliminary data to characterize genetic diversity between and within two *E. tangutorum* populations from Tibet province of China by AFLP analyses [4, 5], until today there is no detailed study has been conducted for molecular characterization using a larger germplasm collection.

Genetic diversity in natural plant populations is held especially by the spatio-temporal environmental heterogeneity, i.e., genetic differentiation is strongly influenced through isolation by distance or local adaptation [17–19]. To estimate the impact of environmental factors on the genetic diversity is indispensable to gain a deeper insight into such evolutionary forces [20–22]. Thus, the combination analysis of molecular markers and eco-geographical data can provide beneficial information for taking up suitable strategies for utilization and conservation of wild plant germplasms [19, 23, 24].

**Fig. 1:**
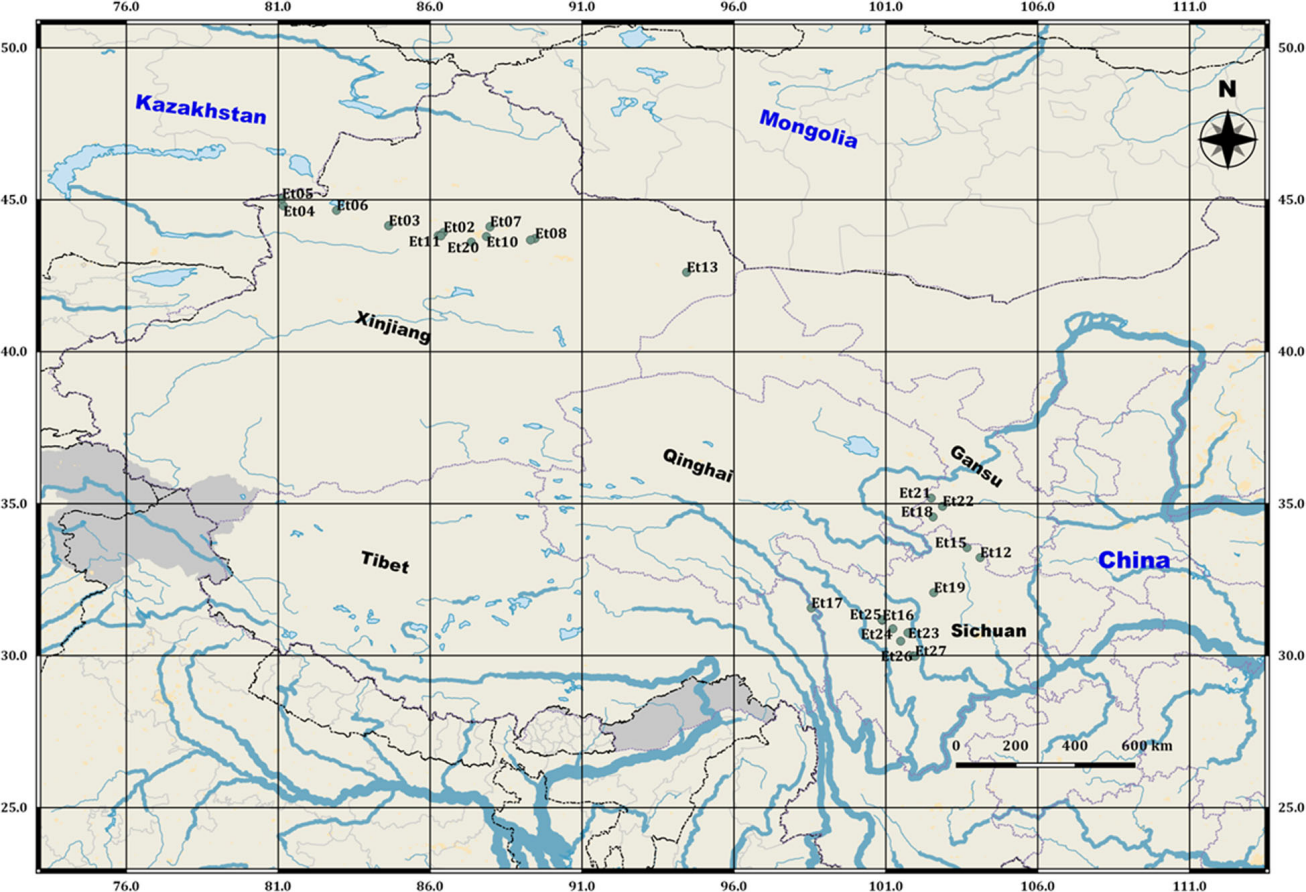
Geographical locations of the studied *Elymus tangutorum* accessions in western China

Here, AFLP markers were used to analyze, on a regional scale, genetic diversity and structure of 27 wild *E. tangutorum* germplasm accessions indigenous from two contrasting climatic zones of Western China, namely Qinghai-Tibet plateau and Xinjiang province (Fig. 1). The goals of the current study were (1) to evaluate and compare the genetic diversity and population structure of *E. tangutorum* germplasm from different areas, and (2) to verify the potential influence of spatial and environmental factors on the patterns of detected population structure.

## Methods

### Plant materials and DNA extraction

Twenty seeds of each accession of *E. tangutorum* were germinated in vermiculite under identical conditions (25 °C, 300 μmols·m^2^·s^− 1^; 16-h photoperiod) in a plant growth chamber. Genomic DNA was extracted from bulked young leaves from five plants per accession employing a CTAB procedure [25]. The quality and quantity of extracted DNA was assayed by 1% agarose gel electrophoresis and NanoDrop® spectrophotometer and was diluted with sterile distilled water to give a final concentration of 100 ng/μL.

### AFLP analysis

AFLP fingerprinting was carried out following Vos et al. (1995) with minor modifications [26, 27], but the primers were labeled with 6-FAM fluorescent dye at the 5’end. Briefly, 300 ng of total genomic DNA was digested using the two restrictive enzymes EcoRI and MseI, following ligation of proper adapters to the restricted DNA fragments. After ligation reactions, pre-selective amplification was performed with (EcoRI + 1)/(MseI + 1) primers. Then the products from the previous step were applied to selective amplification using (EcoRI + 3)/(MseI + 3) selective primers. Initially, 48 selective primer combinations were screened on six accessions, and 14 pairs were chosen based on total number of fragments detected, fragment robustness and polymorphism (Additional file 1: Table S1). The automated separation and detection were fulfilled on an ABI 3730xl sequencer (Applied Biosystems) with the assistance of GeneScan 500 ROX as internal size standard. Genemarker 2.2 (SoftGenetics) was used to score fragments of 60–500 bp with a peak height above or equal to 100 reflective fluorescent units (RFUs). Then AFLP data from all primers was transferred into a binary matrix, scored as“1” (presence of fragment) and “0” (absence of fragment).

### Data analysis

The discriminatory power of each AFLP primer combination was evaluated by calculating polymorphic information content (PIC), marker index (MI), and resolving power (RP) with polymorphic bands only. The formula to calculate PIC value of each primer combination is: PIC_*i*_ = 2 *f*_*i*_ (1 − *f*_*i*_), where PIC_*i*_ is the PIC of *i*th marker, *f*_*i*_ stand for the frequency of the present fragments and 1 − *f*_*i*_ represent the frequency of absent fragments [28]. Marker index (MI) was calculated as MI = PIC × EMR, where EMR (effective multiple ratio) is defined as the product of the proportion of polymorphic loci (PP) and the number of polymorphic loci (NPF), namely the total number of polymorphic fragments per primer [29]. The resolving power (RP) of each primer was estimated as *Rp* = ∑Ib, where Ib (fragment informativeness) defined as *Ib* = 1–2 × |0.5 − p|, where, *p* is the proportion of accessions in which the fragment is present [30].

To understand the genetic relationship among all 27 accessions, pair-wise genetic distance (GD) were calculated on the basis of the Dice’s coefficient by NTSYS-pc v2.10 software. To obtain a more detailed view of the distribution of genetic variation within and between different geographical groups (XJ vs QTP), mean GD among accessions belonging to the same and/or different groups was also calculated. Based on GD matrix, the principal coordinate analysis (PCoA) and Unweighted Pair Group Method with Arithmetic (UPGMA) clustering analysis were performed. The robustness of the dendrograms was assessed with 1000 bootstrap iterations by Freetree software [31]. The Bayesian model-based clustering method was applied to explore the population structure within the studied accessions or genetically diverse population (or groups) using the STRUCTURE software v.2.3.4 (http://web.stanford.edu/group/pritchardlab/structure.html). Following the suggestion by Forsberg et al. [32] for outcrossing species, the admixture model was adopted and the heterozygous loci were treated as missing data. Population structure analysis was performed on AFLP data of all 27 accessions without any prior classification based on the Bayesian model-based method. Evanno’s ad hoc ΔK statistic was used to determine the most probable clustering number of subgroups [33]. We set the number of clusters (K) from 1 to 5 (10 times for each K) for accurate assignments of accessions. For each run, the burn-in period of 50,000 iterations and a run length of 500,000 MCMC replications were implemented. Considering that the indication of the most likely number of groups, namely LnP(D) usually plateaus or increases slightly after the “right K” is reached [25], we used Structure Harvester online software (http://taylor0.biology.ucla.edu/struct_harvest/) to detect optimum value of K based on ΔK method [33]. The output files for the selected K based on ten independent runs were then processed in CLUMMP [34] using the LargeKGreedy algorithm and the G’pairwise similarities. Results were exported to DISTRUCT [35] for better graphical presentation. We also executed spatial population analysis using TESS 2.3.1 [36], which has been described as carrying high percentage of correct inference than other Bayesian clustering algorithm under certain conditions [37]. Geographical coordinates of each accession were contained in AFLP dataset as prior information, and the MCMC algorithm was run under BYM admixture model with 50,000 sweeps and10 000 burn-in. Fifty independent iteration analyses were performed at each value of clusters K (2~5).The value of K with the highest likelihood was determined according to the deviance information criterion (DIC), and the results of runs with 20% lowest DIC values were processed using CLUMMP and DISTRUCT, respectively, as did in the STRUCTURE analysis. Tess outputs was further visualized in a geographical perspective using an R script (POPSutilities) written by Flora Jay [38].

To validate the genetic variation among and within inferred subgroups according to geographic origins or STRUCTURE results, analysis of molecular variance (AMOVA) based on PhiPT (analogue of *F*_*ST*_) value was using GenAlEx 6.5 was performed. The observed (Na) and effective number (Ne) of alleles, expected heterozygosity (H_E_), Shannon’s information index (I) [39], and Nei’s genetic distance among geographic groups were also calculated by GenAlEx.

Based on geographical location of each accession, the corresponding environmental data (precipitation and temperature parameters) was obtained by DIVA-GIS (version 5.2.0.2, http://swww.diva-gis.org/). The geographic and environmental distance matrix was calculated based on the Euclidean distance between pairs of collection sites using NTSYS-pc v2.10. Correlation between geographical and genetic distances (Nei-Li coefficients) between accessions was measured by Mantel test with 9999 permutations using IBD (Isolation by Distance) software v1.53 [40]. A similar analysis was conducted to compare the genetic distance and environmental distance (altitude, precipitation and temperature) for accessions in different geographic groups.

## Results

### Marker informativeness

The AFLP fingerprinting was performed on 27 wild *E. tangutorum* accessions using 14 primer combinations. A total of 554 scorable fragments were detected with a mean of 40 per assay unit, of which 509 (91.7%) were polymorphic and 70 (12.6%) were unique (Tables 1 and 2). The size range of fragments scored across accessions was 60 to 500 bp. The total number of amplified fragments (TNF) per primer pair varied from 30 to 47 and the number of polymorphic fragments per primer pair (NPF) ranged from 26 (E46M51, E-ATT/M-CCA) to 41 (E43M55, E-ATA/M-CGA). The number of unique fragments per primer pair (NFU) ranged from one (for E46M60) (NUF = 1) to 10 (for E40M59 and E50M72). The percentage polymorphism varied from 80.95 to 97.56% with an average of 91.77% per primer combination. This result suggests that *E. tangutorum* germplasm from western China could be characterized well by above AFLP dataset retaining abundant variation. Polymorphism information content (PIC) of each primer pair being 0.5 for AFLP as the dominant marker, 38% of polymorphic loci (195) possessed PIC values above 0.25, implying high discriminatory power of AFLP markers for *E. tangutorum* germplasm. In addition, the marker index (MI) and resolving power (RP) values for individual primer pair were recorded. There were positive correlations between PIC, RP and MI (*r* = 0.98, 0.90 and 0.87, respectively). The most discriminatory primer combinations among 14 primers were E-ATG/M-CTC (E45M60, PIC = 0.3355, MI = 32.73, RP = 20.74) followed by E-AAC/M-GAC (E32M64, PIC = 0.3099, MI = 29.32, RP = 17.63).

**Table 1:** The eco-geographic description on collection sites of *Elymus tangutorum* accessions examined in AFLP analysis

No.	Accessions	Collecting regions	Latitude (N)	Longitude (E)	Altitude (m)	Geographical group	Average annual precipitation (mm)	Average annual temperature (°C)
Et01	PI 598528	Xinjiang, China	43°49′59″	86°16′0″	1665	XJC	243	2.66
Et 02	PI 598530	Xinjiang, China	43°55′59″N	86°25′59″	1040	XJC	191	6.85
Et 03	PI 598537	Xinjiang, China	44°8′59″N	84°37′59″	1620	XJC	220	2.66
Et 04	PI 598552	Xinjiang, China	44°47′59″N	81°9′59″	1700	XJC	392	2.05
Et 05	PI 598554	Xinjiang, China	45°1′59″N	81°7′0″	1300	XJC	343	3.73
Et 06	PI 598555	Xinjiang, China	44°38′59″ N	82°55′0″	300	XJC	105	8.21
Et 07	PI 598558	Xinjiang, China	44°7′0″ N	87°58′0″	1680	XJC	199	7.24
Et 08	PI 598561	Xinjiang, China	43°43′59″ N	89°`27′0″	1400	XJC	175	3.73
Et 09	PI 598564	Xinjiang, China	43°40′59″ N	89°`17′59″	1870	XJC	204	1.18
Et 10	PI 598566	Xinjiang, China	43°47′59″ N	87°`50′59″	1600	XJC	238	4.68
Et 11	PI 610896	Xinjiang, China	43°49′0″ N	86°20′59″	1510	XJC	234	3.60
Et 12	PI 619518	Sichuan, China	33°13′59″ N	104°5′59″	2100	SCC	54	7.93
Et 13	PI 619524	Xinjiang, China	42°37′6″ N	94°26′22″	1750	XJC	228	5.26
Et 14	PI 619570	Sichuan, China	30°28′43″ N	101°29′5″	3500	SCC	698	6.00
Et 15	PI 619571	Sichuan, China	33°33′37″ N	103°40′10″	2480	SCC	773	4.73
Et 16	PI 619572	Sichuan, China	31°10′0″ N	100°52′59″	3520	SCC	669	4.45
Et 17	PI 619588	Sichuan, China	31°34′6″ N	98°32′32″	3600	SCC	652	6.40
Et 18	PI 636678	Gansu, China	34°33′48″ N	102°33′5″	2950	GSC	560	5.00
Et 19	PI 639860	Sichuan, China	32°4′42″ N	102°34′22″	3280	SCC	606	2.16
Et 20	PI 655139	Xinjiang, China	43°36′55″ N	87°21′11″	1320	XJC	841	2.78
Et 21	PI 655187	Gansu, China	35°11′9″ N	102°29′25″	2830	GSC	563	1.95
Et 22	PI 655189	Gansu, China	34°54′48″ N	102°51′47″	2900	GSC	586	2.00
Et 23	PI 655200	Sichuan, China	30°45′6″ N	101°43′31″	2320	SCC	694	8.54
Et 24	PI 655203	Sichuan, China	30°53′12″ N	101°13′40″	3280	SCC	698	5.58
Et 25	PI 655204	Sichuan, China	31°11′22″ N	100°51′50″	2990	SCC	651	6.49
Et 26	PI 655212	Sichuan, China	29°59′35″ N	101°53′0″	3110	SCC	930	4.09
Et 27	PI 655214	Sichuan, China	29°58′59″ N	101°57′24″	2700	SCC	905	5.26

*XJC* Xinjiang province of China (XJC), *SCC* Sichuan province of China, *GSS* Gansu province of China

**Table 2:** Genetic diversity statistics of 14 polymorphic primer pairs used for AFLP fingerprinting of *E. tangutorum* accessions

Primer code	TNF	NPF	NUF	PP(%)	PIC	MI	Rp	I	He
E32M64	37	37	3	97.37	0.3099	29.32	17.63	0.427	0.277
E40M59	42	39	10	92.86	0.2120	19.69	12.37	0.357	0.229
E41M64	42	40	4	95.24	0.2629	25.04	14.59	0.409	0.267
E42M54	30	28	3	93.33	0.2253	21.03	9.63	0.428	0.280
E42M56	38	36	9	94.74	0.2457	23.28	13.11	0.346	0.222
E43M55	47	41	8	87.23	0.2462	21.48	16.15	0.391	0.261
E45M60	41	40	4	97.56	0.3355	32.73	20.74	0.449	0.296
E46M51	31	26	5	83.87	0.2189	18.36	9.56	0.335	0.218
E46M60	42	34	1	80.95	0.1973	15.97	11.04	0.364	0.238
E46M72	40	39	4	97.50	0.3037	29.61	17.63	0.420	0.269
E47M65	41	39	4	95.12	0.2614	24.86	15.04	0.376	0.239
E48M54	45	39	2	86.67	0.2198	19.05	13.26	0.414	0.273
E50M59	40	34	3	85.00	0.2347	19.95	13.63	0.436	0.295
E50M72	38	37	10	97.391	0.2325	22.64	12.96	0.385	0.249
Total	554	509	70	–	–	–		0.400	0.260
Minimum	30	26	1	80.95	0.1973	15.97	9.56	0.335	0.218
Maximum	47	41	10	97.56	0.3355	32.73	20.74	0.449	0.296
Mean	39.64	36.36	5	91.77	0.2504	23.07	14.10	0.396	0.258

*TNF* total number of fragments, *NPF* number of polymorphic fragments, *PP* the fraction of polymorphic loci, *PIC* polymorphic information content, *MI* marker index, *RP* resolving power, *NUF* number of unique fragments, *NRF* number of rare fragments

### Population structure analysis

**Fig. 2:**
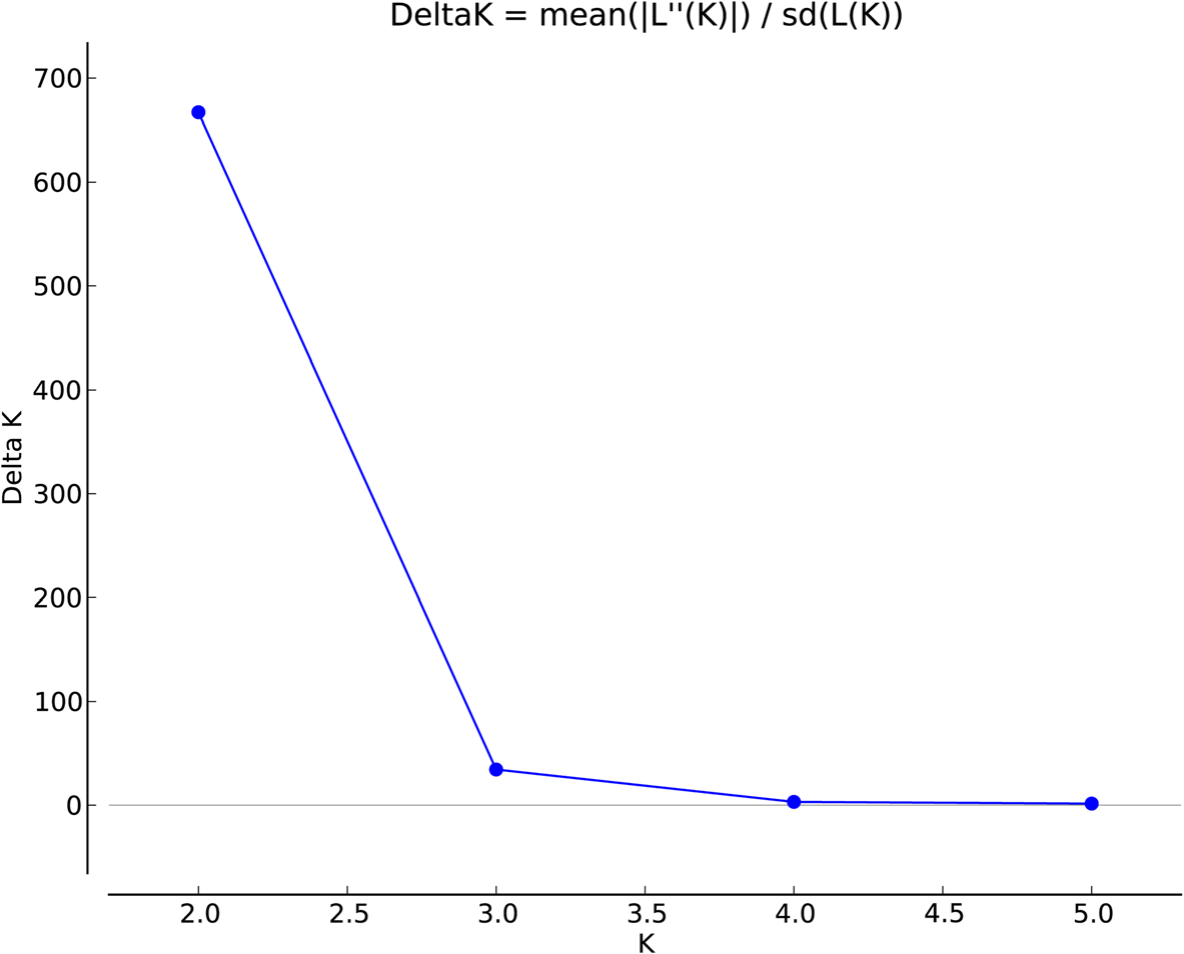
Estimation of number of subpopulations (K) for the studied panel from STRUCTURE analysis

**Fig. 3:**
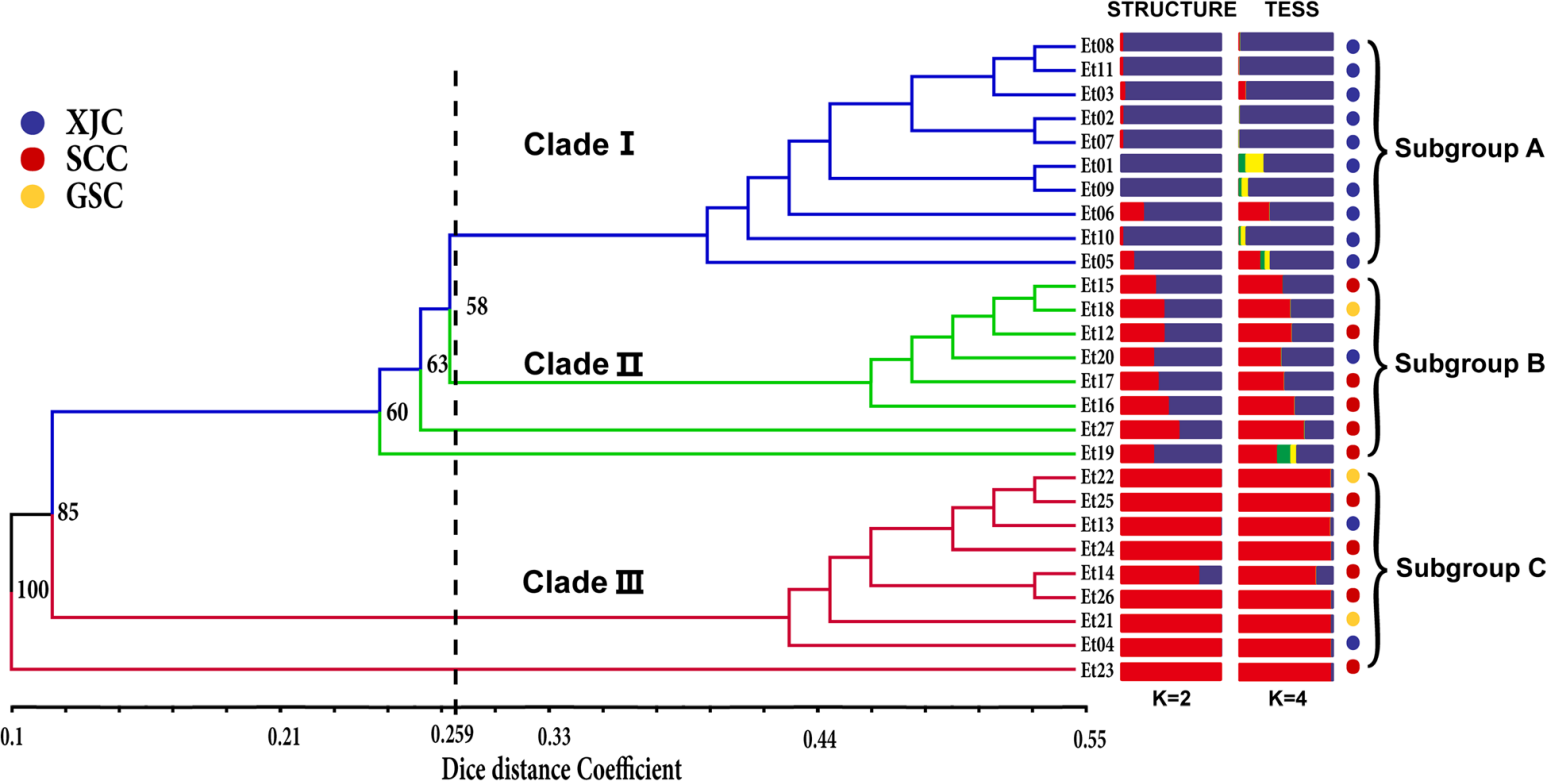
Dendrogram of 27 *Elymus tangutorum* accessions based on Dice’s coefficient using UPGMA method by NTSYS-pc v2.10 software and genetic structure using a Bayesian analysis by STRUCTURE v.2.3.4 and TESS 2.3.1 software

The results of population using the STRUCTURE software showed a clear peak of the ΔK values when K was set at 2 (Fig. 2),which indicate that the sampled accessions belonged to two inferred genome fraction (Fig. 3). Samples with a inferred genome fraction value (membership proportion) of 0.8 or more to a subgroups are considered as pure, and less than 0.80, as admixture [32]. Considering individual’s membership proportion (Q_i_), all 27 accessions were assembled into three Bayesian subgroups designated as subgroup A (pure, blue fraction ≥0.8), subgroup B (admixture, 0.2 ≤ blue fraction ≤0.8) and subgroup C (red fraction ≥0.2). The three subgroups included 10, 8 and 9 accessions, respectively. Geographically, most of accessions from Xingjiang (XJC) (10, 71.43%) belonged to subgroup A and the rest resided in subgroup B (Et20) and C (Et04 and Et13). Six accessions from Sichuan (SCC) were part of subgroup B and the five remaining accessions were part of subgroup C. For the three accessions from Gansu (GSC), Et18 consisted in subgroups B and the remaining two belonged to subgroup C.

The Bayesian clustering results by TESS 2.3.1 had lowest DIC values (highest probability) at K_max_ = 4, indicating that the sampled accessions belonged to four inferred genome fraction (Additional file 2: Figure S1). This pattern is slightly different from the result from Structure runs in which the optimal K = 2. However, Considering the proportion of individual’s membership (Qi), the Bayesian subgroups based on TESS was almost exactly equivalent to the Structure result, both of which have the two dominant ancestry coefficients (Fig. 3). On the other hand, the geographic map displaying the Q matrix spatially by R script [38] also exhibited a general trend that the samples from XJC is obviously different from the samples from QTP, although the number of collection sites were limited and the spatial mapping for each accession is modeled over the whole Asia area rather than just considered at actual sampling sites (Additional file 3: Figure S2).

### Cluster and principal coordinate analysis

Based on the 509 polymorphic fragments, estimates of the genetic distances (Dice’s coefficient) ranged from 0.1024 to 0.7137 with an average of 0.2773, implying a largely genetic divergence in the germplasm collections studied. Accessions Et08 and Et11 were most related due to a minimum genetic distance of 0.1024, while Et01 and Et23 were most divergent with the maximum genetic distance of 0.7137. The rest of accessions have exhibited different middle levels of genetic distances. Compared with 0.2611 of inter-group dissimilarity, the accessions from Xinjiang were more homogenous with lower dissimilarity values (0.2214), whereas the QTP accessions were more variable genetically with higher dissimilarity values (0.2785). These results were also confirmed by STRUCTURE analysis that most of Xinjiang accessions had pure type of genome fraction. The genetic distance matrix was used to build a hierarchical dendogram of genetic relationships based on the UPGMA clustering method (Fig. 3), in which three major clades were clearly categorized with further internal subgroupings. These three clades were identified at the 0.259 dissimilarity level. Grouping of most of accessions was congruent with their respective geographical origins. However, the accession Et20 and Et13 from Xinjiang did not separate clearly from QTP accessions. The bootstrap support of all major branches were higher than 50%, and a majority of them ranged from 70 to 99% revealing reliability of data and clustering results. Despite some differences, there was coherence between Bayesian method and hierarchical clustering analysis (Fig. 3). The UPGMA dendrogram could separate well three Bayesian subgroups, except for the accessions Et27, Et19 and Et23 were revealed as outliers.

**Fig. 4:**
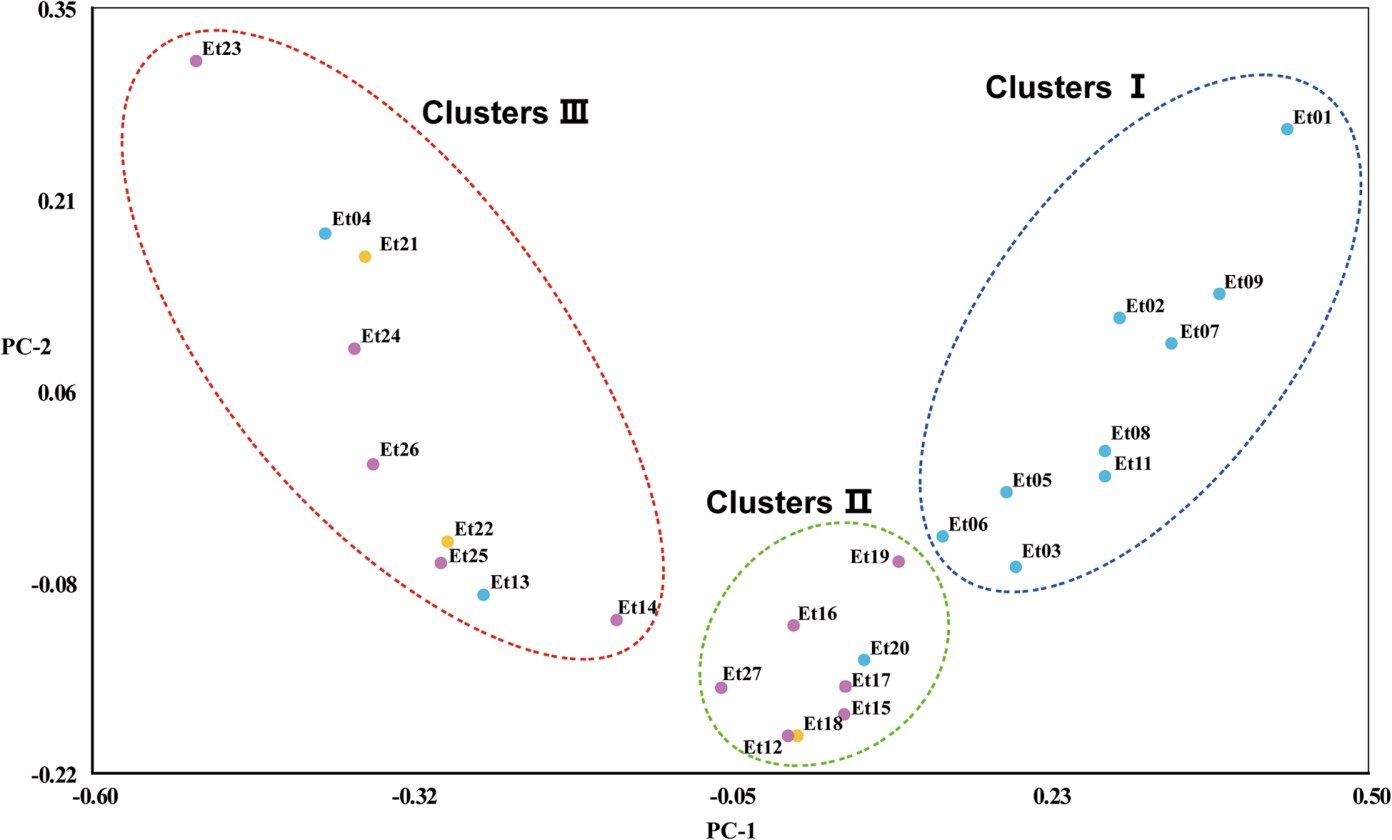
Principal component analysis of 27 *Elymus tangutorum* accessions based on AFLP data

A similar clustering patter of the studied germplasm was demonstrated by the principal coordinate analysis (PCoA) (Fig. 4) illustrating that scatter-plot dipiction of PCoA is comparable to either of the hierarchical and Bayesian cluster analysis. That is, most of studied accessions were separated according to their geographical groups and/or the locations sites then put in the same cluster. PCoA was performed based on Nei-Li distances and confirmed division of 27 accessions into three major clusters: ClusterI, II and III. The first two principal coordinates represented the total of 69.1% of the total molecular variation (60.25, and 8.65%, respectively). The first axis (PC1), which contributed 60.25% of the variance, separated most of accessions, which have been assigned to three UPGMA clusters. The second axis (PC2),explained 8.65% of the variation, could further distinguish the accessions from cluster I and III.

### Genetic diversity estimates

Geographically, the region of Gansu and Sichuan Province are both part of the QTP region. Hence, we merged the GSC and the SCC group into a new group named QTP group. The total gene diversity across different ecogeographical regions was estimated based on 509 polymorphic fragments (Additional file 4: Table S2). The percentage of polymorphic loci (PP) was found to be higher in accessions of QTP (73.29%) than those of Xinjiang (70.76%). The Xinjiang group (XJC) showed high Nei’s indices of gene diversity (He) of 0.24 and Shannon’s diversity (I) of 0.36, whereas QTP group displayed a moderate levels of diversity (He = 0.21and I = 0.32). For the three Bayesian subgroups, the P*P* value was found to be highest among accessions from subgroup A (67.69%) followed by subgroup C (65.16%). The markers were least informative for assessment of genetic variability of accessions from subgroup B with PP restricted to 53.25%. Similarly, subgroup A showed the highest intra-group diversity (I = 0.36 and He = 0.24), whereas subgroup B had the lowest intra-group diversity (I = 0.01 and He = 0.0.01).

### AMOVA analysis

The two independent AMOVA analyses implemented relying on geographic origins and STRUCTURE clustering results and a highly significant variation (*P* < 0.01) was detected (Table 3). For regional groups, 16.24% of the total variation was due to differences among populations and 73.90% variation was due to divergence within groups. Both of the pairwise *F*_*ST*_ values of two regional groups were significant (*P* ≤ 0.01) which were tested by GenAlex 6.5. The total *F*_*ST*_ = 0.162 indicated that the genetic variability is moderate between QTP and XJC groups. The three subgroups inferred from STRUCTURE showed 81 and 19% of total variance within and among the groups. This means that the total *F*_*ST*_ was 0.188 (*P* ≤ 0.01) and the derived three subgroups are highly structured by Bayesian inference panel. Significant differences in the F_ST_ were observed for all the pairwise comparisons of three subgroups inferred from STRUCTURE analysis (Table 4). The maximum pairwise *F*_*ST*_ (0.274) found between subgroup A and subgroup C was confirmed by their great difference of inferred genome fraction (Table 4). In addition, AMOVA analysis indicate that the level of gene flow either between geographic areas or between Bayesian clusters was low and insufficient to make homogenization in the *E. tangutorum* collections of western China.

**Table 3:** Analysis of molecular variance (AMOVA) among and within geographical groups and subgroups derived from STRUCTURE

Groups	Source of variation	d.f.	Sum of squares	Variance components	Percentage of variation (%)	Fst	*P*-value
Two Geographical Groups (XJC, QTP)	Among groups	1	235.476	12.636	16.24%	0.162	≤0.01
	Within groups	25	1628.154	65.126	83.75%		
Three Bayesian subgroups (A, B, C)	Among groups	2	380.382	14.324	19%	0.188	≤0.01
	Within groups	24	1483.247	61.802	81%

**Table 4:** Pairwise estimates of Fst values between Bayesian subgroups of *E. tangutorum* accessions as inferred by STRUCTURE software

	Subgroup A	Subgroup B	Subgroup C
Subgroup A	–	–	–
Subgroup B	0.130*	–	–
Subgroup C	0.274**	0.110**	–

The P value for estimated Fst was calculated using 10,000 permutations (*P < 0.05; **P < 0.01)

### Mantel test

To verify the eco-geographical factors influencing genetic structuring, Mantel’s test was used to estimate matrix correlations between genetic distance and geographical, elevation, average annual temperature, and average annual precipitation distances. A weak but significant pattern of isolation by geographic distance was detected within all accessions (*r* = 0.312; *P* = 0.001). A similar result was found in each group except GSC (Table 5). This pattern derives largely from measurable differentiation among geographic groups seen in STRUCTURE and UPGMA clustering analysis. Furthermore, there was a faint or non-significant correlation between distance matrices of genetic and environmental factors for all accessions. However, a significantly positive correlation was demonstrated between genetic distance and the average annual precipitation distance in XJC (*r* = 0.685; *P* = 0.01) and Subgroup A (*r* = 0.594; *P* = 0.05) (Table 5). In addition, relatively high Mantel r-value were observed between environmental factors and genetic distance for Gansu accessions, all of which were non-significant due to a small group size (only 3 accessions).

**Table 5:** Correlation between genetic distance and eco-environmental distance based on pairwise Mantel test for all accessions, geographical groups and Bayesian subgroups

Groups	Factors	r	p
All	Geographical distance	0.312	0.001
	Elevation	0.118	0.13
	Average annual precipitation	0.285	0.01
	Average annual temperature	0.187	0.13
XJC	Geographical distance	0.313	0.076
	Elevation	−0.052	0.4
	Average annual precipitation	0.685	0.01
	Average annual temperature	−0.051	0.36
SCC	Geographical distance	0.113	0.187
	Elevation	0.3	0.09
	Average annual precipitation	−0.005	0.69
	Average annual temperature	0.439	0.11
GSC	Geographical distance	0.809	0.32
	Elevation	0.971	0.24
	Average annual precipitation	0.92	0.16
	Average annual temperature	0.309	0.49
QTP	Geographical distance	0.131	0.19
	Elevation	0.325	0.1
	Average annual precipitation	0.05	0.32
	Average annual temperature	0.365	0.05
Subgroup A	Geographical distance	0.399	0.07
	Elevation	0.226	0.21
	Average annual precipitation	0.594	0.05
	Average annual temperature	0.014	0.51
Subgroup B	Geographical distance	−0.125	0.5
	Elevation	−0.064	0.41
	Average annual precipitation	0.208	0.24
	Average annual temperature	0.024	0.37
Subgroup C	Geographical distance	−0.032	0.53
	Elevation	0.054	0.4
	Average annual precipitation	−0.186	0.31
	Average annual temperature	0.079	0.28

*XJC* Xinjiang province of China (XJC), *SCC* Sichuan province of China, *GSS* Gansu province of China

## Discussion

### AFLP polymorphism and discriminating capacity of the assays

The practical availability of molecular markers in plant germplasm characterization depends on their power to discriminate the genotypically different accessions analyzed. In the present paper, the efficiency of AFLPs were assessed by recording different marker parameters concerning polymorphism, PIC (Polymorphic Information Content), MI (marker index) and RP (resolving power). In total, 509 AFLP polymorphic fragments with 91.77% of polymorphism were observed in the current work, which was obviously higher than previous works by AFLP in *E. tangutorum* and other related *Elymus* species. In the studies of *E. dahuricus* complex diversity, 69.35, 58.79, 29.65 and 27.14% of polymorphic fragments for *E.excelsus*, *E.dahuricus*, *E.woroschilowii* and *E. tangutorum* was obtained respectively [5]. In three *Elymus* species indigenous to East Asia, 34.2, 30.1 and 51.5% of polymorphism was detected for *E. tsukushiensis*, *E. humidus* and *E. dahuricus* respectively [14]. So far, the primer combinations used in this study successfully established high levels of genetic distinctness among the studied *E. tangutorum* accessions using AFLP fingerprints. The level of polymorphism obtained here with AFLP markers is also higher than that reported for other *Elymus* germplasm in western China by other types of dominant markers such as *E. sibiricus* by ScoT (89%) [13] and *E. nutans* by ISSR (91.4%) [41]. In all, the participation of polymorphic fragments largely relies on the species, origin ecogeographic area and the differentiation degree of sampled accessions, amount of primers and even type of molecular marker used [42, 43]. Moreover, as all primer combinations produced unique fragments (on average, 5 per primer), those primers could be used to differentiate particular accessions [44].

The polymorphic information content (PIC) values of 0.25 in current study demonstrated good marker discriminatory power in view of PIC value starting from 0 to 0.5 for dominant marker like AFLPs [45, 46]. This values also agree with AFLP studies done with other forage grass such as *Dactylis glomerata* [47] and *Phalaris aquatica* [48].The Marker index (MI) and resolving power (RP), two importantly alternative parameters in selecting informative markers for diversity studies [49, 50], had also displayed high efficiency of AFLP markers in unveiling polymorphisms in *E. tangutorum* germplasm. A very strong and positive correlation observed among PIC, RP and MI value in present work indicates the use of any of three parameters to screen the informative primer combinations [38]; although the relationship between the informativeness of molecular markers for identifying germplasms and these marker parameters is not totally clear [46]. Primer combinations E-ATG/M-CTC and E-AAC/M-GAC should be the most informative due to the highest records on PIC, RP and MI. Therefore, they are recommended for use in germplasm diversity analyses of other *E. tangutorum* collections. The Shannon index is an accurate alternative measure of diversity due to no need for estimate of allele frequencies under Hardy-Weinberg equilibrium. The Shannon diversity detected in this study (0.34) was higher than those of *E. sibiricus* germplasm from western China by SCoT (*I* = 0.285) and EST-SSR (*I* = 0.237) markers [13, 51], also indicating a high level of genetic diversity in the accessions of *E. tangutorum* (Table 2).

### Clustering pattern and genetic structure

It is attractive for breeders and germplasm curators to know the width of genetic diversity within a plant species of great importance [52]. The spatial genetic diversity analysis was conducted to verify the relationship between *Elymus tangutorum* accessions and biogeographical patterns by different clustering approaches. The UPGMA tree, the PCoA scatter plot, and the STRUCTURE analyses revealed a similar or identical membership among 27 wild *E. tangutorum* accessions, with a general grouping pattern including three genetically distinct and consistent groups. When these accessions are grouped according to clusters distinguished(Fig. 3), a greater range of Nei-Li distances is observed, which is comparable to previous studies for other *Elymus* germplasm with similar collection areas [13, 53, 54]. The first cluster derived from UPGMA tree consisted of ten accessions from Xinjiang Province in southwest of China, having a typical temperate continental climate. Meanwhile, Cluster I had the distinctly pure membership based on Bayesian clustering. The second cluster was primarily formed by accessions from southeast of Qinghai-Tibet Plateau (QTP), a region greatly far from Xinjiang, having a typical plateau mountain climate. Cluster II was corresponding well to the admixture subgroup STRUCTUE by analysis. The third cluster was also chiefly comprised by the accessions from southeast of QTP, with another distinctly pure membership. In short, the above results were in agreement with the geographical proximity of studied accessions and their genetic relationships.

Exceptions to this pattern of clustering were three accessions (Et04, Et13 and Et20) from Xinjiang,which were grouped with QTP accessions in Clade II and Clade III respectively. Since *E. tangutorum* is used in natural grassland restoration projects [6], it is possible that these three accessions are not native accessions from QTP and might have been introduced through domestic trade of seeds from Xinjiang. By contrasting the figures of UPGMA, PCoA and STRUCTURE analyses, two accessions (Et19 and Et27) assigned to Bayesian Subgroup II, fall into outgroups of UPGMA analyses. The possible reason causing such relationships may be STRUCTURE assumes that the loci within a population are under Hardy–Weinberg equilibrium (HWE) and it is susceptible to many factors such as non-random mating, genetic drift, mutations, gene flow and selection [55]. Nonetheless, in different clustering analyses, it was largely found that separate clusters were often obtained in accordance with the respective geographic origin of accessions. Therefore the cluster pattern in present work might be due, in part, to differences in the adaptation of ecotypes to various selection forces from local conditions [56]. These results are in agreement with earlier studies which showed that geographical separation of other *Elymus* species germplasm did not always result in greater genetic differentiation [12, 57].

Indeed, the genetic diversity and population structure are determined by joint effects of many factors including geographic distribution, life cycle, mating system, selection and adaptation [58]. Considering the genetic differences among studied regions, the accessions from Xinjiang province showed higher diversity (I = 0.24, He = 0.36) than those from QTP (I = 0.21, He = 0.32). With regard to the Bayesian subgroups, higher diversity was found among accessions in subgroup A, which correspond to accessions from Xinjiang province. Based on a membership probability threshold of 0.80, most accessions (66.67%) such as subgroup A and C appeared to have a pure population ancestry in their genome component, which might result from limited gene flow among wild accessions with distantly geographical origin [59]. This explains why we have observed high diversity within geographical groups. However, more than half of the accessions from QTP were composed of admixture of genome fraction. Such case could be supported by the fact that *E. tangutorum* was frequently used to grassland restoration in Qinghai-Tibetan Plateau as mentioned above [6, 60]. Therefore, human activities may lead to frequently natural crossing by wind pollination between adjacent stands.

Results of AMOVA analysis showed low genetic variation among regions accounted for only 16.2% of the germplasm collection, whereas within-region variation accounts for 83.8% of the total variability. Similarly, only 19% of variation was detected for Bayesian groups,. These results on variation among accessions within geographical groups were confirmed with previous studies for *E. nutans* and *E. sibiricus* collections with similar origins [12, 57]. However, our results are more extreme. This could be a consequence of high gene flow resulting from grassland restoration project or the limited number of studied accessions for each region. The low among-group variance components indicated that the genetic background attributable to the geographical origin contributes slightly to the observed genetic diversity. Besides, the high gene flow and differentiation within *E. tangutorum*, supported by clustering and AMOVA, was also probably driven by its mating system, which is one of the important life-history feature that strongly influence genetic diversity and population structure [61]. As reported in genetic diversity study for *Elymus dahuricus* complex [5], *E. tangutorum* is probably a predominantly self-pollinated species with 0.9 of *F*_*ST*_ value. In their review, Hamrick and Godt [62] claimed that short-lived, self-pollinating species have most of their genetic diversity partitioned among populations rather than within populations. Similarly, Nybom [58] assumed that short-lived self-pollinating species allocate most of their genetic variability within populations. Each *E. tangutorum* accession in present investigation could be treated as a population consisting of seeds of neighboring individual plants collected from differently distant locations [63]. This situation may limit gene flow between accessions by geographic isolation barriers (i.e. mountains, rivers), thus the variation will maintain its characteristics within each accession. Although the within-accession variability is not determined by individual-based analysis, we have indeed observed the high degree of differentiation within this species according to UPGMA and PCoA analysis based on bulked DNA samples. Hence it is not surprising that the vast proportion of AFLP variation detected in *E. tangutorum* was present rather within than among groups, since the large-scale geographical group contained numerous accessions with distinctly genetic architecture. These results could be confirmed by that the totally average genetic distance (0.277) between all of accessions was higher than that (0.261) between accessions from the two main geographical groups (QTP and Xinjiang).

### Correlation of genetic diversity and environmental variables

In virtue of the evolutionary forces, the decisive factor to influence the maintenance of genetic variability is the spatial variation of environment and the ecological difference between habitats [24]. Remarkably, the Mantel test between genetic distance and geographical distance in all *E. tangutorum* accessions and most groups was weakly correlated (Table 5) and similar results were reported in previous studies of *Elymus* species [2, 57]. Within accessions from QTP group, we found lower *r*-values of geographical distance (*r* = 0.131) compared with the XJC group (*r* = 0.313), which was not significantly different from zero and might be related to the highly heterogeneous topography nature of the QTP collection. The complex topographic features and climate will work together to form varieties niches to influence the genetic variability [64]. Thus, spatial distribution cannot be solely accounted for by a simple isolation-by-distance model and requires additional factors that influence the observed genetic population structure, such as environmental factors [24]. In the Mantel test between genetic distance and environmental distance, we found a significantly positive correlation between genetic distance and average annual precipitation (*r* = 0.685; *P* = 0.01 and *r* = 0.594; *P* = 0.05) in the XJC group and Bayesian subgroup A. A possible explanation for this results is that in Xinjiang with arid and/or semi-arid temperate climate, the lower precipitation hinders seed germination and lead to decrease plants’ genetic diversity [17]. In the diversity study of *E. nutans* germplasm from western China, the environmental divergence like elevation is also related to genetic distance (*r* = 0.695; *P* = 0.01) [41]. On the other hand, the relationship between environmental factor and genetic distance is difficult to be fully resolved in complex environment [18]. This could be responsible for the weak and non-significant correlation between genetic distance and environmental distance found in QTP group. Besides, although the accessions of GSC group had high Mantel r-value (Table 5) between genetic distance and environmental distance except the average annual temperature (*r* = 0.309), the correlation between the genetic diversity and environmental factor was still unraveled due to the small population size.

## Conclusions

This study indicates that AFLP markers are a powerful tool to measure genetic diversity in *E. tangutorum* and geographical and environmental factors (especially precipitation) together plays a crucial role in genetic differentiation patterns. These findings highlight the importance of local adaptation in shaping patterns of genetic structure inferred in *E. tangutorum* accessions fromWestern China. Therefore, collecting and assessing *E. tangutorum* germplasm from major geographic regions and special ecogeographic environment such as Qinghai-Tibet Plateau, will help to expand the genetic base and sample the full extent of available variation.

## Additional files

Additional file 1: **Table S1.** Adaptor and primer sequences used for AFLP analysis. (DOCX 17 kb)

Additional file 2: **Figure S1.** Bayesian clustering results for 27 accessions of *Elymus tangutorum* using the program TESS from K = 2 to K = 5. (TIF 686 kb)

Additional file 3: **Figure S2.** Maps representing the mean membership proportions for K clusters of *Elymus tangutorum. (TIF 80 kb)*

Additional file 4: **Table S2.** Different genetic diversity estimates for geographical groups and Bayesian subgroups of *E. tangutorum* based on AFLP results. (DOCX 18 kb)

## Abbreviations

AFLP: Amplified fragment length polymorphism
AMOVA: Analysis of molecular variance
CTAB: Hexadecyl trimethyl ammonium Bromid
GD: Genetic distance
I: Shannon information index
ISSR: Inter-simple sequence repeat
MI: Marker index
NPF: Number of polymorphic loci
PCR: Polymerase chain reaction
PIC: Polymorphism information content
PcoA: Principal coordinate analysis
RAPD: Random Amplified Polymorphic DNA
RP: Resolving power
SCoT: Started codon targeted polymorphism
SRAP: Sequence—related amplified polymorphism
SSR: Simple sequence repeat
TNF: Total number of amplified fragments
UPGMA: Unweighted pair-group method with arithmetic means
