# Supplementary material for: Genetic diversity and structure of Elymus tangutorum accessions from western China as unraveled by AFLP markers
Source: Hereditas. 2019 Jan 29;156:8. doi: 10.1186/s41065-019-0082-z (PMC6352457; doi:10.1186/s41065-019-0082-z)
Supplement: Supplementary file 1 — Table S1. Adaptor and primer sequences used for AFLP analysis. (DOCX 17 kb) [file 41065_2019_82_MOESM1_ESM.docx]

**Additional file 1: Table S1**

Adaptor and primer sequences used for AFLP analysis

| Adaptor and primer | Code | sequence (5'-3') |
| --- | --- | --- |
| EcoR I | EcoR I-B-F | CTCGTAGACTGCGTACC |
|  | EcoR I-B-R | AATTGGTACGCAGTCTAC |
| Mse I | Mse I-B-F | GACGATGAGTCCTGAG |
|  | Mse I-B-R | TACTCAGGACTCAT |
| Pre-amplification |  |  |
| EcoR I +0 | EcoR I-A | GACTGCGTACCAATTC |
| Mse I +0 | Mse I-A | GATGAGTCCTGAGTAA |
| Selective primers |  |  |
| EcoR I +3 |  |  |
| E32 | E-AAC | GACTGCGTACCAATTCAAC |
| E39 | E-AGA | GACTGCGTACCAATTCAGA |
| E40 | E-AGC | GACTGCGTACCAATTCAGC |
| E41 | E-AGG | GACTGCGTACCAATTCAGG |
| E42 | E-AGT | GACTGCGTACCAATTCAGT |
| E43 | E-ATA | GACTGCGTACCAATTCATA |
| E44 | E-ATC | GACTGCGTACCAATTCATC |
| E45 | E-ATG | GACTGCGTACCAATTCATG |
| E46 | E-ATT | GACTGCGTACCAATTCATT |
| E47 | E-CAA | GACTGCGTACCAATTCCAA |
| E48 | E-CAC | GACTGCGTACCAATTCCAC |
| E50 | E-CAT | GACTGCGTACCAATTCCAT |
| Mse I +3 |  |  |
| M51 | M-CCA | GATGAGTCCTGAGTAACCA |
| M54 | M-CCT | GATGAGTCCTGAGTAACCT |
| M55 | M-CGA | GATGAGTCCTGAGTAACGA |
| M56 | M-CGC | GATGAGTCCTGAGTAACGC |
| M59 | M-CTA | GATGAGTCCTGAGTAACTA |
| M60 | M-CTC | GATGAGTCCTGAGTAACTC |
| M64 | M-GAC | GATGAGTCCTGAGTAAGAC |
| M65 | M-GAG | GATGAGTCCTGAGTAAGAG |
| M72 | M-GGC | GATGAGTCCTGAGTAAGGC |
| M83 | M-TCA | GATGAGTCCTGAGTAATCA |
