# Supplementary figures and images for: Genetic diversity and structure of Elymus tangutorum accessions from western China as unraveled by AFLP markers
Source: Hereditas. 2019 Jan 29;156:8. doi: 10.1186/s41065-019-0082-z (PMC6352457; doi:10.1186/s41065-019-0082-z)

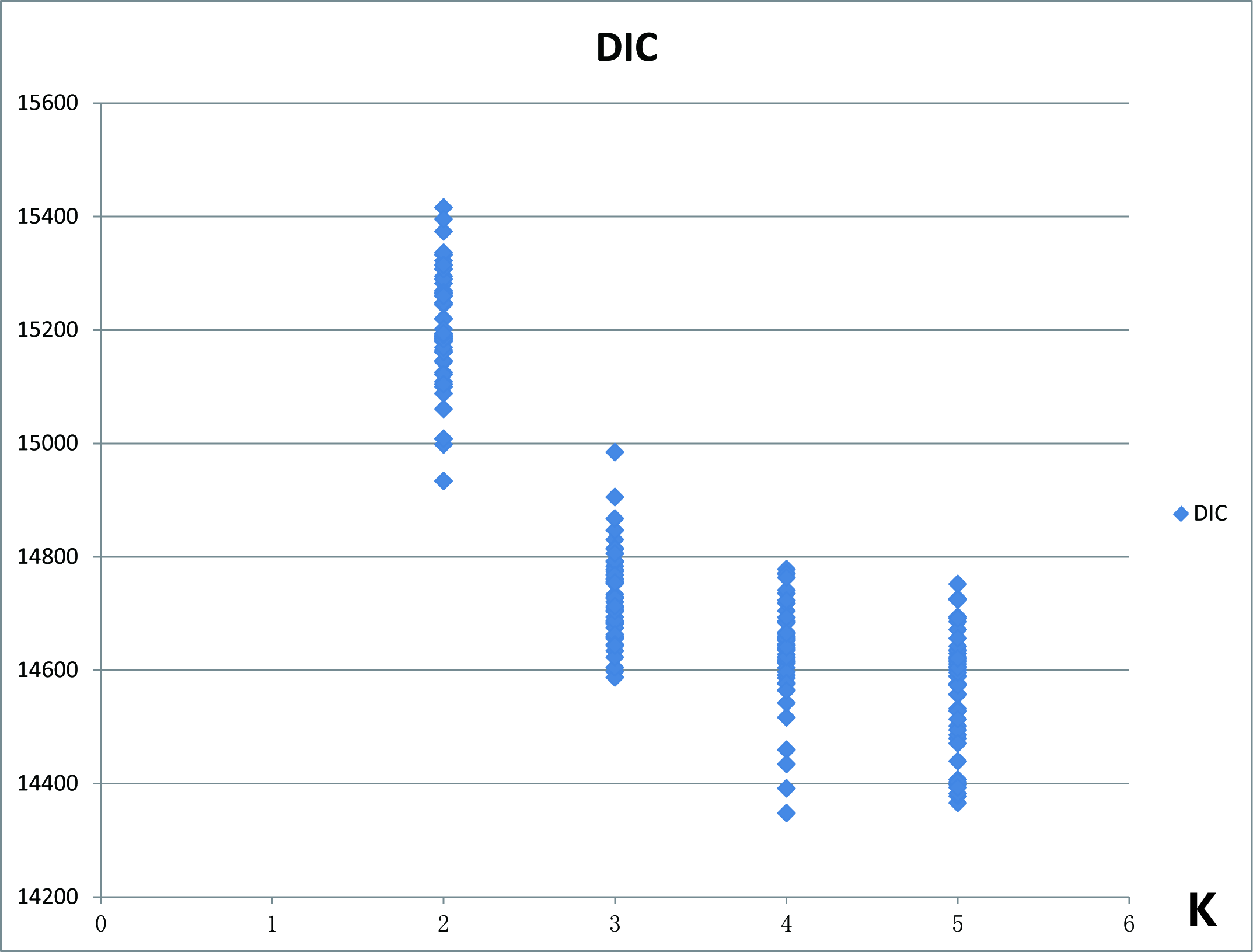

Supplement: Supplementary file 2 — Figure S1. Bayesian clustering results for 27 accessions of Elymus tangutorum using the program TESS from K = 2 to K = 5. (TIF 686 kb) [file 41065_2019_82_MOESM2_ESM.tif]

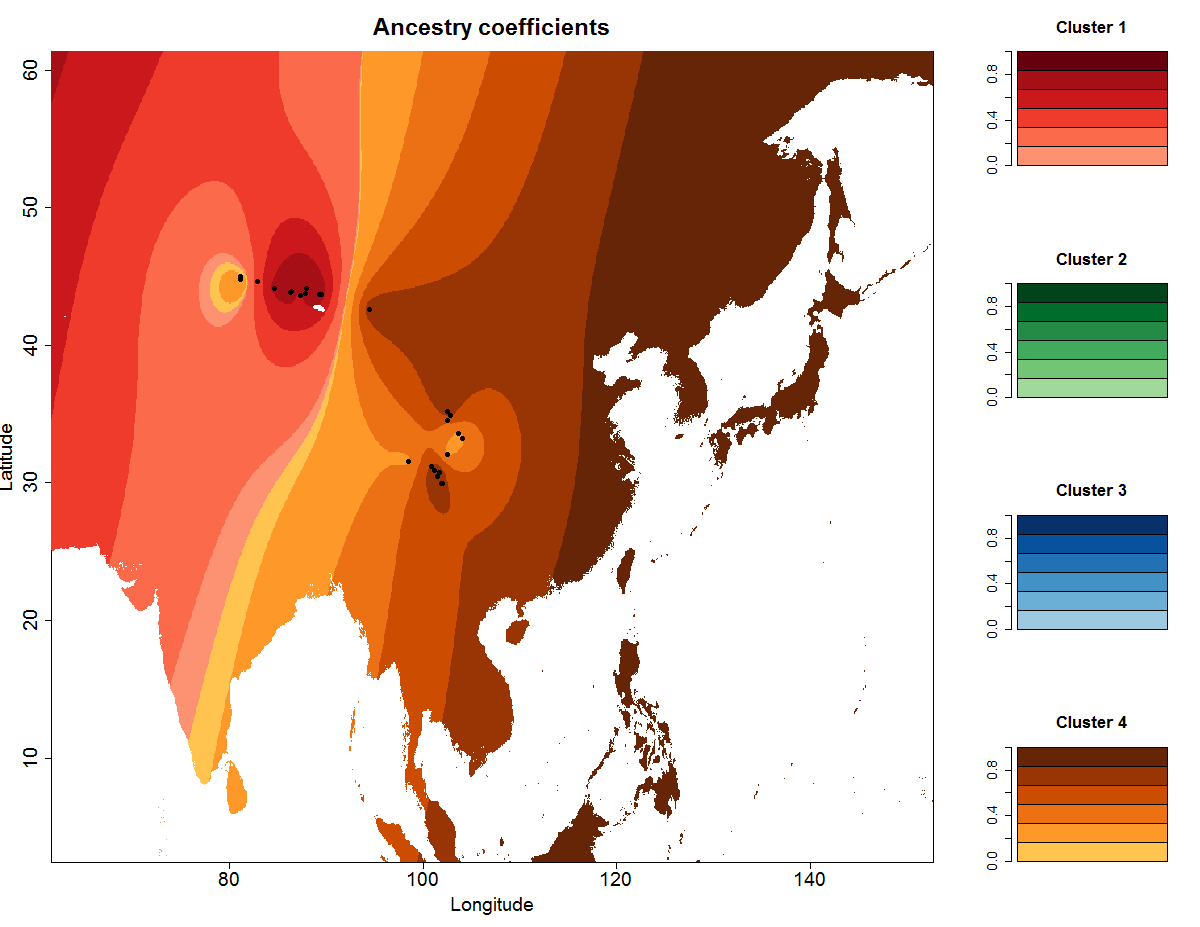

Supplement: Supplementary file 3 — Figure S2. Maps representing the mean membership proportions for K clusters of Elymus tangutorum. (TIF 80 kb) [file 41065_2019_82_MOESM3_ESM.tif]
