# Supplementary material for: Genetic diversity and structure of Elymus tangutorum accessions from western China as unraveled by AFLP markers
Source: Hereditas. 2019 Jan 29;156:8. doi: 10.1186/s41065-019-0082-z (PMC6352457; doi:10.1186/s41065-019-0082-z)
Supplement: Supplementary file 4 — Table S2. Different genetic diversity estimates for geographical groups and Bayesian subgroups of E. tangutorum based on AFLP results. (DOCX 18 kb) [file 41065_2019_82_MOESM4_ESM.docx]

**Additional file 4: Table S2**

Different genetic diversity estimates for geographical groups and Bayesian subgroups of *E. tangutorum* based on AFLP results

| Groups | | N | Na | Ne | I | He | PP(%) |
| --- | --- | --- | --- | --- | --- | --- | --- |
| Three Geographical Groups | SCC | 13 | 1.59±0.029 | 1.42±0.017 | 0.36±0.012 | 0.24±0.009 | 70.76 |
|  | XJC | 11 | 1.53±0.033 | 1.37±0.016 | 0.33±0.012 | 0.22±0.008 | 70.4 |
|  | GSC | 3 | 0.83±0.035 | 1.16±0.013 | 0.15±0.010 | 0.1±0.007 | 27.07 |
|  |  |  |  |  |  |  |  |
| Two Geographical Groups | XJC | 13 | 1.59±0.029 | 1.42±0.017 | 0.36±0.012 | 0.24±0.009 | 70.76 |
|  | QTP | 14 | 1.58±0.031 | 1.35±0.015 | 0.32±0.011 | 0.21±0.008 | 73.29 |
|  |  |  |  |  |  |  |  |
| Three Bayesian subgroups | Subgroup A | 10 | 1.54±0.031 | 1.43±0.017 | 0.36±0.012 | 0.24±0.009 | 67.69 |
|  | Subgroup B | 8 | 1.316±0.034 | 1.284±0.015 | 0.256±0.012 | 0.168±0.008 | 53.25 |
|  | Subgroup C | 9 | 1.32±0.035 | 1.28±0.015 | 0.26±0.011 | 0.17±0.008 | 65.16 |

Na: number of different alleles; Ne: number of effective alleles; I: Shannon's information index; He: expected heterozygosity ; PP: percentage of polymorphic loci
